# Ocular fundus examination in emergency medicine: an Australian survey of equipment, training, current practice, and clinician perceptions

**DOI:** 10.1038/s41433-026-04844-z

**Published:** 2026-09-17

**Authors:** Yujie Wang, Wenyi Hu, Raphael Chang, Li Li, Sam Freeman, Carmel Crock, Franz E. Babl, Gary Ayton, Lucia Le-Kim, Hamed Akhlaghi, Honghua Yu, Tissa Wijeratne, Zongyuan Ge, Myra B. McGuinness, Peter Van Wijngaarden, Mingguang He, Zhuoting Zhu

**Affiliations:** 1 Centre for Eye Research Australia, Royal Victorian Eye and Ear Hospital, Melbourne, VIC Australia; 2 Ophthalmology, Department of Surgery, The University of Melbourne, Melbourne, VIC Australia; 3 Department of Medicine, The University of Melbourne, Melbourne, VIC Australia; 4 Emergency Department, St Vincent’s Hospital (Melbourne) Limited, Fitzroy, VIC Australia; 5 Australian Institute of Health Innovation, Macquarie University, Sydney, NSW Australia; 6 Department of Critical Care, Faculty of Medicine, Dentistry and Health Sciences, University of Melbourne, Melbourne, VIC Australia; 7 Emergency Department, The Royal Victorian Eye and Ear Hospital, East Melbourne, VIC Australia; 8 Emergency Department, The Royal Children’s Hospital, Melbourne, VIC Australia; 9 Murdoch Children’s Research Institute, Melbourne, VIC Australia; 10 Department of Paediatrics, The University of Melbourne, Melbourne, VIC Australia; 11 Department of Emergency Medicine, Sunshine Hospital, Western Health, St Albans, VIC Australia; 12 Faculty of Health, Deakin University, Burwood, VIC Australia; 13 Guangdong Eye Institute, Department of Ophthalmology, Guangdong Provincial People’s Hospital (Guangdong Academy of Medical Sciences), Southern Medical University, Guangzhou, China; 14 Joint Shantou International Eye Center of Shantou University and the Chinese University of Hong Kong, Shantou, China; 15 Department of Neurology, Sunshine Hospital, Western Health, St Albans, VIC Australia; 16 Department of Public Health and Psychology, La Trobe University, Bundoora, VIC Australia; 17 AIM for Health Lab, Faculty of Information Technology, Monash University, Clayton, VIC Australia; 18 Airdoc-Monash Research, Monash University, Clayton, VIC Australia; 19 Centre for Epidemiology and Biostatistics, Melbourne School of Population and Global Health, The University of Melbourne, Melbourne, VIC Australia; 20 The Florey Institute of Neuroscience and Mental Health, University of Melbourne, Melbourne, VIC Australia; 21 School of Optometry, The Hong Kong Polytechnic University, Hong Kong SAR, China; 22 Research Centre for SHARP Vision, The Hong Kong Polytechnic University, Hong Kong SAR, China; 23 Centre for Eye and Vision Research, Hong Kong SAR, China

**Keywords:** Health services, Diagnosis

## Abstract

**Objectives:**

Fundus examination is critical for the early detection of serious ocular and neurological conditions in the emergency department (ED), yet it remains underused. This study aimed to investigate the current practice of fundus examination among ED doctors in Australia.

**Methods:**

A national cross-sectional online survey was distributed to the Australasian College for Emergency Medicine members. The 28-item questionnaire assessed demographics, equipment availability, training, confidence, practice patterns, perceived barriers and image-interpretation performance using five representative fundus images. Descriptive statistics and logistic regression were used for analysis.

**Results:**

A total of 135 emergency clinicians completed the base items (58.5% female; median experience, 10 years [interquartile range 5.5–20]; 25.9% from regional/remote areas). Most respondents had access to direct ophthalmoscopes (94.5%) or slit lamps (63.7%), while a few had retinal cameras (10.4%). Although 42.2% clinicians had received related training in EDs, over half of them receiving it less than once every five years. While 82.1% of clinicians considered fundus examination important, there was poor agreement on appropriate indications, and only 50.8% reported performing it themselves when indicated. Only 26.5% doctors expressed confidence in performing and interpreting fundus examinations. Image-interpretation accuracy across images was 44.5% (95% CI, 38.5–50.7%). Doctors most reported low confidence, insufficient training as barriers, while enhanced training and portable cameras were the key suggested enablers.

**Conclusion:**

Underutilisation of fundus examination in EDs highlights gaps in training, standardised indications, confidence, image-interpretation performance and equipment usability. Addressing these barriers is key to improving neuro-ophthalmic triage and early detection of sight-threatening and life-threatening conditions.

## Introduction

The ocular fundus examination is vital to detect vision-threatening disease and provides a direct, non-invasive window onto the central nervous system and systemic vasculature[1]. In the emergency department (ED), it holds critical diagnostic insight for patients with neurological or visual symptoms, enabling early detection of vision- or life-threatening conditions and guiding timely management [2–5].

Despite its clinical significance, fundus examination remains underutilised in the ED[6, 7]. For instance, the FOTO-ED study revealed that only 14% of patients presenting with headache received fundoscopy in ED at a U.S. academic centre [8]. Contributing factors likely include the technical demands of traditional techniques and limited skills and confidence among non-ophthalmic clinicians; however, systematic evaluation of these barriers within EDs is limited.

Traditional direct ophthalmoscopy, though long relied upon, is limited by its narrow field of view, technical difficulty and low diagnostic yield when performed by non-ophthalmic clinicians [9]. In recent years, alternative fundus imaging technologies, such as retinal cameras, are gaining attention as more user-friendly tools in acute care[1, 4, 5, 8, 10].. Despite these advancements, the current availability of such devices and corresponding training remains poorly understood.

Several surveys from the United Kingdom (UK) [11, 12], the United States (US) [13] and Canada [14] have reported that many emergency clinicians feel undertrained and lack confidence in diagnosing and managing ophthalmic emergencies. However, no national-level data exist from Australia. Moreover, to date, no study has systematically explored emergency clinicians’ perceptions of fundus examination alongside their diagnostic performance on key fundus findings.

To address these gaps, we conducted the first national survey of Australian emergency doctors to characterise current fundus examination practices. This study investigated the availability of equipment, training exposure, practice pattern, clinician confidence and diagnostic performance among Australian emergency doctors.

## Materials and methods

### Study design and distribution

This cross-sectional study was conducted using an online survey administered via convenience sampling. Ethical approval was granted by the Human Research Ethics Committee of St Vincent’s Hospital Melbourne, Australia (reference: LRR 229/24).

The target population comprised medical doctors currently working in a patient-facing roles within EDs across Australia. The survey was implemented using REDCap (Research Electronic Data Capture), a secure web-based platform hosted by the Centre for Eye Research Australia, Melbourne, Australia [15, 16].

To facilitate participant recruitment, support was obtained from the Australasian College for Emergency Medicine (ACEM), the peak professional body for emergency medicine training and certification in Australia and New Zealand. With ACEM’s endorsement, the survey was promoted through its official weekly Bulletin and distributed to members. Additionally, ED directors from multiple hospital sites were invited to assist with survey dissemination via departmental communication channels.

The survey invitation included a link to a plain language statement and a participant information and consent form, outlining the study’s purpose, procedures and data confidentiality provisions. Informed consent was implied through voluntary participation. Upon confirming consent, participants were automatically directed to the survey. Data collection occurred between December 2024 and May 2025. One week prior to the survey’s closure, reminder emails were sent to individuals who provided consent but had not yet completed the questionnaire.

### Questionnaire

The survey questions were developed in collaboration with experts in ophthalmology and emergency medicine, who provided professional feedback to ensure the questionnaire was tailored to the clinical context of ED doctors and capable of effectively capturing current practice. The final version is included in Supplementary File 1.

The questionnaire consisted of 28 items. Eligibility screening was embedded in the survey entry to confirm respondents met the inclusion criteria. The survey covered several domains, including respondent and institutional demographics, availability of ophthalmic equipment, training in fundus examination, clinical practice patterns and attitudes and image interpretation. Questions regarding fundus exam training details were conditionally displayed only to participants who reported prior training experience with fundus examination in the ED. The final section assessed emergency clinicians’ ability to identify sight-threatening or life-threatening retinal manifestations using five representative color fundus photographs, each illustrating a distinct retinal condition that may present in the ED: optic disc edema, central retinal artery occlusion, retinal vein occlusion, retinal hemorrhage and a normal fundus. Participants were asked to select the single most appropriate fundus finding or associated diagnosis for each image.

### Statistical analysis

The analysis included respondents who met eligibility criteria and completed at least the respondent and institutional demographics section (items 1-11). For specific sections, analyses were conducted on participants with complete responses for the variables of interest.

Categorical variables were summarised as frequencies and percentages. Continuous variables were assessed for normality using the Shapiro-Wilk test. As years of ED experience were non-normally distributed, they were summarised as median and interquartile range (IQR). Free-text responses under ‘Other’ categories were reviewed and thematically grouped. Frequently cited responses were recoded and incorporated into the descriptive analysis, while more detailed or context-rich comments were synthesised to inform qualitative interpretation. Participants’ institutional locations were categorised as self-reported ‘Major City’ or ‘Regional/Remote’ for subgroup comparisons. Inter-rater agreement regarding indications for fundoscopy was evaluated using Fleiss’ kappa, which assesses the consistency of clinicians’ judgments. Negative κ values indicate agreement less than expected by chance.

For the image interpretation task, the proportion correct for each image was defined as the proportion of participants who selected the correct response, calculated only among those who completed the task. Overall image-interpretation accuracy was calculated at the image-response level by aggregating all participant responses across the five images. Proportions are reported with 95% confidence intervals (CIs) using the Wilson method. Overall image-interpretation accuracy and 95% CIs were estimated using robust standard errors to account for clustering of responses within clinicians. Univariable binomial logistic regression with robust standard errors clustered at the participant level was used to investigate factors associated with accurate interpretation of fundus images. Variables examined included participant gender, specialist emergency medicine training level, practicing region, approach to performing fundus examination, confidence and perceived importance of fundus examination and prior fundus examination training received in the ED. We reported odds ratios (ORs) with corresponding 95% CIs. Statistical significance was defined as a two-sided *P* value ≤ 0.05. No correction for multiple testing was applied as these analyses are considered exploratory.

All analyses were conducted using R statistical software (version 4.3.3; R Foundation for Statistical Computing).

## Results

### Participants characteristics

A total of 181 ED doctors initiated the survey. Of these, 172 completed the eligibility screening and provided consent. Following the exclusion of respondents who did not meet the inclusion criteria or who provided incomplete demographic information, 135 participants were included in the survey analysis (Supplementary Figure 1). Of the included participants, 79 (58.5%) were female and the median (IQR) of work experience in EDs was 10 (5.5–20) years (Table 1). The majority of respondents were either ACEM Fellows or Overseas Trained Emergency Specialists (*n *= 91, 67.4%), followed by Trainee Registrars in emergency medicine (*n* = 30, 22.2%). Among respondents, 100 (74.1%) were working in major cities and 132 (97.8%) in public hospitals.

**Table 1:** Participant demographics.

	Participant Characteristic	Major city (*N* = 100)	Regional/remote area (*N* = 35)	Total (*N* = 135)
**Gender**	Female	55 (55.0%)	24 (68.6%)	79 (58.5%)
	Male	45 (45.0%)	10 (28.6%)	55 (40.7%)
	Prefer not to say	0 (0.0%)	1 (2.9%)	1 (0.7%)
**ED training**	Emergency medicine fellowship/Overseas trained emergency medicine specialist	67 (67.0%)	24 (68.6%)	91 (67.4%)
	Trainee registrar in emergency medicine	22 (22.0%)	8 (22.9%)	30 (22.2%)
	Medical doctor without additional training in emergency medicine	11 (11.0%)	3 (8.6%)	14 (10.4%)
**Practice Year**	5 or less	27 (27.0%)	7 (20.0%)	34 (25.2%)
	6-10	28 (28.0%)	8 (22.9%)	36 (26.7%)
	11-15	14 (14.0%)	9 (25.7%)	23 (17.0%)
	16 or more	31 (31.0%)	11 (31.4%)	42 (31.1%)
**Hospital setting**	Public	98 (98.0%)	34 (97.1%)	132 (97.8%)
	Private	2 (2.0%)	1 (2.9%)	3 (2.3%)
**Number of doctors in ED**	20 or less	19 (19.0%)	17 (48.6%)	36 (26.7%)
	21–40	42 (42.0%)	8 (22.9%)	50 (37.0%)
	41–60	29 (29.0%)	5 (14.3%)	34 (25.2%)
	61 or more	10 (10.0%)	5 (14.3%)	15 (11.1%)
**Number of patients per day in ED**	100 or less	4 (4.0%)	11 (31.4%)	15 (11.1%)
	101-200	37 (37.0%)	11 (31.4%)	48 (35.6%)
	201-300	48 (48.0%)	10 (28.6%)	58 (43.0%)
	301 or more	11 (11.0%)	3 (8.6%)	14 (10.4%)

*ED* emergency department.

### Equipment availability

**Fig. 1:**
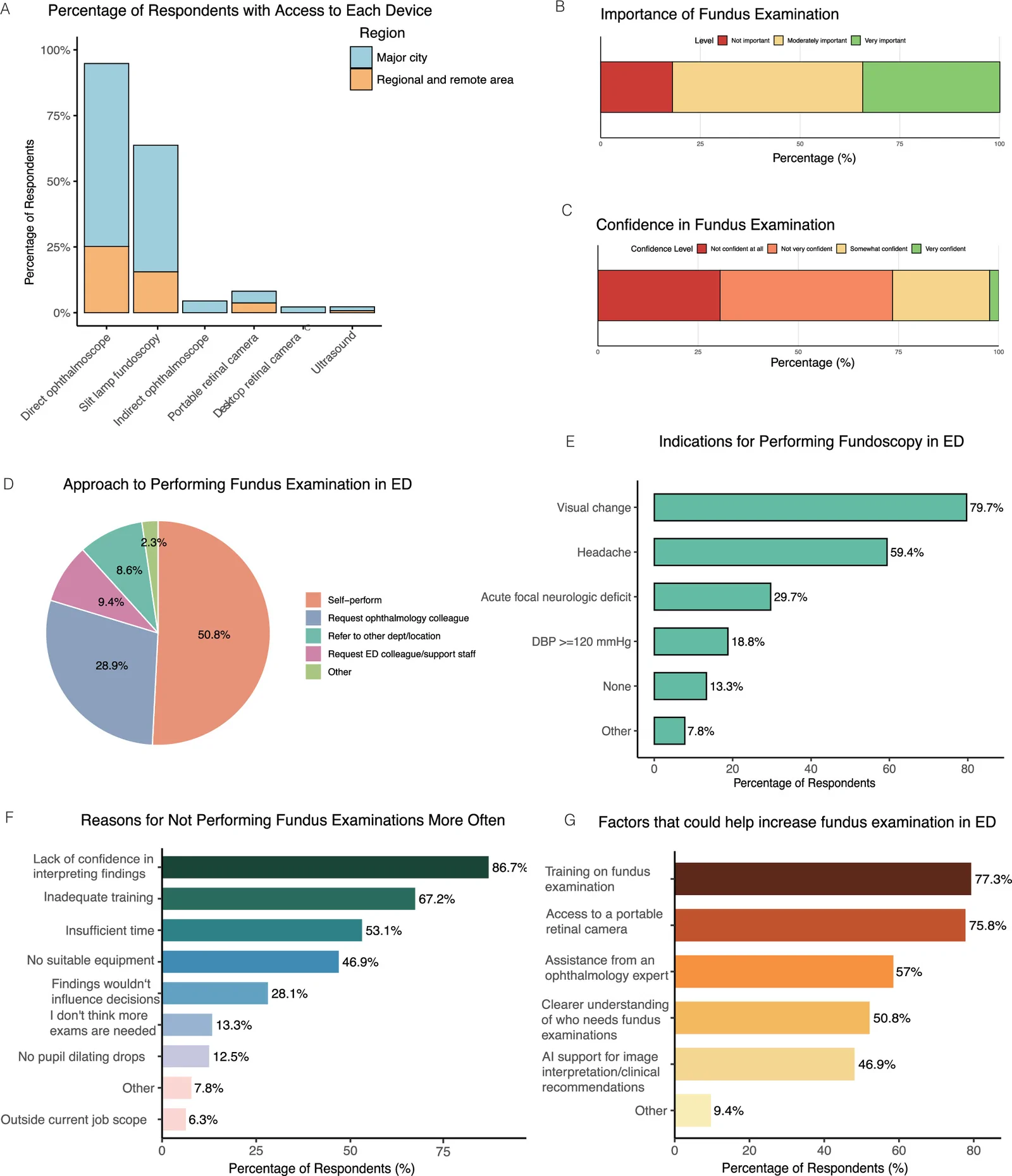
Emergency clinicians’ access to fundus examination equipment, clinical practices, and perceptions in the emergency department (ED). **A** Proportion of respondents reporting access to various fundus examination devices in their ED, stratified by practice location, based on the question: ‘Which fundus/retinal examination equipment is readily accessible in your emergency department?’. **B** Perceived importance of being able to perform fundus examinations in the ED, based on: ‘Do you think it is important for ED doctors to be able to perform fundus examination?’. **C** Self-reported confidence in performing and interpreting fundus examinations, based on: ‘How confident are you in performing and interpreting fundus examinations?’. **D** Reported approaches to conducting fundus examinations in the ED, from: ‘If an ED patient required fundus examination, most often:’ **E** Clinical indications for performing fundoscopy, as reported in response to: ‘Which types of patients would you consider performing/requesting fundoscopy on?’ Multiple responses were allowed. **F** Reported reasons for not performing fundus examinations more often in the ED, from: ‘It has been reported that fundus examination is not routinely conducted in emergency departments. What do you believe are the main reasons fundus examination is not performed more often in your workplace?’ Multiple responses were allowed. **G** Reported factors that could help increase the fundus examination in the ED, from: ‘Which of the following factors do you believe could help you perform fundus examinations more frequently in the emergency department?’ Multiple responses were allowed. Percentages represent the proportion of total respondents selecting each option. Panels B and C display Likert-style response distributions.

The direct ophthalmoscope was reported as the most commonly available device for fundus examination among participants (*n* = 128, 94.8%), followed by slit lamp fundoscopy (*n* = 86, 63.7%) and portable retinal camera (*n* = 11, 8.1%) (Fig. 1A and Table 2). Most respondents reported access to one (*n* = 43, 31.9%) or two (*n* = 81, 60.0%) types of equipment. Among those with access to a single type of device, most (*n* = 37, 86.0%) reported the direct ophthalmoscope as the sole available instrument.

**Table 2:** Available equipment for fundus examination in the emergency department.

		Major city (*N* = 100)	Regional/remote area (*N* = 35)	Total (*N* = 135)
**Accessible equipment**	Direct ophthalmoscope	94 (94.0%)	34 (97.1%)	128 (94.8%)
	Indirect ophthalmoscope	6 (6.0%)	0 (0.0%)	6 (4.4%)
	Slit lamp fundoscopy	65 (65.0%)	21 (60.0%)	86 (63.7%)
	Desktop retinal camera	3 (3.0%)	0 (0.0%)	3 (2.2%)
	Portable retinal camera	6 (6.0%)	5 (14.3%)	11 (8.1%)
	Ultrasound	2 (2.0%)	1 (2.9%)	3 (2.2%)
**Number of accessible equipment**	Three	9 (9.0%)	2 (5.7%)	11 (8.1%)
	Two	59 (59.0%)	22 (62.9%)	81 (60.0%)
	One	32 (32.0%)	11 (31.4%)	43 (31.9%)
Only equipment*	Direct ophthalmoscope	27 (84.4%)	10 (90.9%)	37 (86.0%)
	Indirect ophthalmoscope	1 (3.1%)	0 (0.0%)	1 (2.3%)
	Slit lamp fundoscopy	3 (9.4%)	1 (9.1%)	4 (9.3%)
	Portable retinal camera	1 (3.1%)	0 (0.0%)	1 (2.3%)

Data are presented as number and percentage. Multiple responses were allowed.

*‘Only equipment’ refers to participants who reported access to only one type of equipment (total *n* = 43, major city *n* = 32, regional/remote *n* = 11).

### Training on fundus examination

Less than half of the respondents (*n* = 54, 42.2%) reported that they have received some sort of training in fundus examination in the ED setting and most were trained in direct ophthalmoscopy (*n* = 50, 92.6%). Practical hands-on sessions were the most common way to provide the training (*n* = 45, 83.3%). In addition, training frequency was low, with 31 (57.4%) respondents having received it less than once every five years, and 14 (25.9%) respondents having only a single session (Table 3).

**Table 3:** Training on fundus examination in the emergency department.

		Major city (*N* = 96)	Regional/remote area (*N* = 32)	Total (*N* = 128)
**Training in ED**	Yes	42 (43.8%)	12 (37.5%)	54 (42.2%)
	No	54 (56.3%)	20 (62.5%)	74 (57.8%)
Training* **context**	Direct ophthalmoscope	38 (90.5%)	12 (100.0%)	50 (92.6%)
	Indirect ophthalmoscope	4 (9.5%)	1 (8.3%)	5 (9.3%)
	Slit lamp fundoscopy	21 (50.0%)	3 (25.0%)	24 (44.4%)
	Fundus camera	4 (9.5%)	4 (33.3%)	8 (14.8%)
	Diagnosis	12 (28.6%)	1 (8.3%)	13 (24.1%)
	Other	1 (2.4%)	0 (0.0%)	1 (1.9%)
Frequency*	Annual training	2 (4.8%)	0 (0.0%)	2 (3.7%)
	Every 2 to 3 years	5 (11.9%)	1 (8.3%)	6 (11.1%)
	Every 4 to 5 years	1 (2.4%)	0 (0.0%)	1 (1.9%)
	<once/5 years	22 (52.4%)	9 (75.0%)	31 (57.4%)
	Single session	12 (28.6%)	2 (16.7%)	14 (25.9%)
Training* **method**	Observational learning	8 (19.0%)	1 (8.3%)	9 (16.7%)
	Practical training	34 (81.0%)	11 (91.7%)	45 (83.3%)

ED = emergency department.

^*^‘Training context’, ‘Frequency’ and ‘Training method’ were reported only by those who had received such training (total *n* = 54, major city *n* = 42, regional/remote *n* = 12).

Multiple responses were allowed where applicable.

### Current practice and perspectives

Most respondents regarded the ability of ED doctors to perform fundus examinations as important, with 44 (34.4%) rating it as ‘very important’ and 61 (47.7%) as ‘moderately important.’ (Fig. 1B and Supplementary Table 1). Despite this, only three clinicians (2.3%) reported being very confident and 31 (24.2%) somewhat confident in performing and interpreting fundus examinations (Fig. 1C). When fundus examination was clinically indicated, only half of the respondents (65, 50.8%) reported performing the examination themselves, while almost a third (37, 28.9%) would consult an ophthalmology colleague (Fig. 1D).

Visual changes (*n* = 102, 79.7%) and headache (*n* = 76, 59.4%) were the most frequently selected indications for fundoscopy, whereas fewer selected acute neurologic deficits (*n* = 38, 29.7%) or diastolic blood pressure) ≥120 mmHg (*n* = 24, 18.8%) as indications for retinal examination. Free-text responses under ‘other’ (*n* = 10, 7.8%) included severe hypertension, painful eye, ocular trauma and diabetes as other indications for ophthalmoscopic examination in the ED. Notably, 17 (13.3%) respondents reported no clinical indication under which they would consider fundoscopy (Fig. 1E). There was poor inter-rater agreement on the clinical scenarios warranting fundus examination (Fleiss’ κ = –0.062, *p* = 0.006), indicating variability in clinician judgment regarding appropriate indications. Responses to specific headache presentations also varied substantially. For instance, for headaches with past history of increased intracranial pressure, 25 (19.5%) respondents reported always performing fundoscopy, 41 (32.0%) never and 62 (48.4%) sometimes (Supplementary Fig. 2).

### Barriers and facilitators of performing fundus examination in the ED

The most frequently reported barriers to performing fundus examination in the ED were lack of confidence in interpreting findings (*n* = 111, 86.7%), inadequate training (*n* = 86, 67.2%), insufficient time (*n* = 68, 53.1%) and no suitable equipment (*n* = 60, 46.9%) (Fig. 1F and Supplementary Table 2). In addition, 16 (12.5%) participants reported no pupil-dilating eye drops. Free-text responses also cited a lack of reliable training resources and, when tools were available, limited use due to poor maintenance, missing parts, or lack of bedside availability (Supplementary Tables 3 and 4).

When asked what might facilitate more frequent examination, most respondents endorsed enhanced training (*n* = 99, 77.3%) and access to a portable retinal camera (*n* = 97, 75.8%), followed by support from an ophthalmology expert (*n* = 73, 57.0%), clearer understanding of clinical indications (*n* = 65, 50.8%) and artificial intelligence (AI) support for image interpretation/clinical recommendations (*n* = 60, 46.9%) (Fig. 1G).

### Image interpretation performance and associated factors

Of the 115 respondents who completed the fundus interpretation task, 94 (81.7%) answered at least one question correctly and 16 (13.9%) answered all five correctly. The overall image interpretation accuracy across five fundus images was 44.5% (95% CI: 38.5–50.7%) (Table 4). Performance varied by condition, with the highest proportion of correct responses for retinal artery occlusion (65.2%, 95% CI: 55.8—73.9%) and the lowest for a normal fundus (21.7%, 95% CI: 14.6—30.4%).

**Table 4:** Correct interpretation of fundus images (proportion correct with 95% confidence intervals).

		Correct number/All	Proportion Correct (95% CI)
**Q 1**	**Optic disc oedema**	57/115	49.6% (40.1–59.0%)
**Q 2**	**Retinal artery occlusion**	75/115	65.2% (55.8–73.9%)
**Q 3**	**Retinal vein occlusion**	46/115	40.0% (31.0–49.6%)
**Q 4**	**Retinal haemorrhages**	53/115	46.1% (36.8–55.6%)
**Q 5**	**Normal fundus**	25/115	21.7% (14.6–30.4%)
	**Total**	256/575	44.5% (38.5–50.7%)

Based on individual image performance, each row represents one test item with a single correct answer. The image interpretation accuracy rate was calculated as the proportion of participants who selected the correct diagnosis for each image.

Higher odds of accurate image interpretation were observed among male participants (OR 1.76, *p* = 0.028), those completed specialist emergency medicine training (OR 3.14, *p* = 0.002), those would perform fundus examination themselves (OR 2.08, *p* = 0.004), those who were somewhat confident (OR 2.77, *p* = 0.005) or very confident (OR 13.11, *p* = 0.008) in performing and interpreting fundus examinations (see Supplementary Table 5). Practicing region, prior training in fundus examination within ED and perceived importance of fundus examination were not significantly associated with performance.

## Discussion

This study provides an overview of current fundus examination practices among Australian emergency clinicians. Our study reported that while most clinicians recognize that fundus examination is clinically important in selected cases, a lack of evidence-based guidelines to guide retinal examination, low confidence among ED doctors in performing and interpreting their examinations and inadequate and infrequent fundus examination training have contributed to various practices in the ED, with only half the ED clinicians performing fundoscopy themselves when clinically indicated.

Our research highlights the limited usability and practical barriers of fundus examination equipment in the ED. Our findings are broadly consistent with previous surveys conducted in other countries, where most emergency clinicians also reported access to slit lamps (92.4% in the US; 82.2% in Canada; 79.8% in the UK) and direct ophthalmoscopes (86.6% in the US; 97.3% in Canada; 97.4% in the UK)[12–14]. Direct ophthalmoscopy is technically challenging; it offers a limited 5° field of view and requires a darkened environment or dilated pupils to optimise the view [9]. It has relatively low diagnostic accuracy when performed by non-ophthalmologists [17]. A UK study found that when using a direct ophthalmoscope, emergency doctors had a sensitivity of only 0.31 in detecting abnormal optic discs [18]. Furthermore, the proximity required between clinician and patient during use raises concerns in the context of the COVID pandemic and occupational violence [17, 19]. As for the slit lamp, it is primarily used for examining the ocular anterior segment in some EDs, such as identifying foreign bodies or corneal injuries. Most non-ophthalmology clinicians report a lack of training or access to appropriate fundus lenses to perform fundus examinations. Importantly, most traditional fundoscopy techniques typically require pharmacologic pupil dilation to optimise visualisation, which was identified as another barrier in EDs[20]. Lack of pupil-dilating eye drops and uncertainty about their use in the ED have hindered the utility of dilating drops in the non-ophthalmology emergency settings, especially when both fundus examination and pupillary monitoring are needed [21, 22]. Another factor is concerns about post-dilation functional limitations, like impaired driving, that may unnecessarily increase the length of stay in the ED.

Training in fundus examination also remains limited, with only 42.2% of respondents (54/128) reporting any training within the ED, mostly on direct ophthalmoscopes. Training rates in direct ophthalmoscopy among Australian clinicians appear lower than in the US (65.3%)[13] and the UK (47.1%) [12]. In addition, among trained respondents, 83.3% (45/54) reported training less than once every five years or only a single session. ‘Inadequate training to use available equipment’ was identified by 67.2% of emergency clinicians (86/128) as a key barrier to performing fundus examinations. Overall, 77.3% of respondents (99/128) endorsed enhancing training as a helpful intervention to improve fundus examination uptake. This aligns with prior international studies, where 95.3% of UK emergency doctors would like to see more formal training in the management of eye patients [12], 75.1% of US physicians wished they had received more ophthalmic training during residency [13], and only 35.6% of Canadian physicians felt they had received adequate training in ophthalmoscopy [14]. Our findings further underscore the international need for enhanced ophthalmic training in emergency care.

Our survey revealed that while most emergency clinicians recognise the importance of fundus examination, perceptions of when it is indicated vary considerably. This was reflected by poor inter-rater agreement across clinical scenarios warranting fundus examination (Fleiss’ κ = –0.062), with the negative kappa suggesting agreement worse than chance. Clinically, this indicates substantial inconsistency in deciding when fundus examination should be performed in the ED, which may contribute to variable practice, unnecessary examinations or referrals, and delayed recognition of important ocular findings. For example, although papilledema is a well-known red flag in headache presentations[23, 24], clinicians varied in their views on when to perform fundoscopy for headache. This lack of consensus highlights the need for clear clinical guidance and structured decision-support pathways. Half of respondents (65/128) indicated that a clearer understanding of when fundoscopy is warranted would facilitate its more frequent use. In the time-pressured ED setting, defined criteria are critical to distinguish non-urgent cases from those needing urgent assessment. Besides, we observed that despite recognizing the clinical indications for fundus examination, many emergency clinicians refrain from performing it themselves, likely due to limited confidence in ophthalmoscopic skills or a preference to refer patients to ophthalmologists. This practice may delay diagnosis and increase reliance on specialist consultations. Therefore, establishing a streamlined and ED-oriented clinical pathway is critical. Recent studies have explored ED pathways using non-mydriatic fundus photography to screen patients presenting with acute vision loss, headache, or neurologic signs [14, 25–28], confirming shorter ED stays and fewer ophthalmology referrals [28].

‘Lack of confidence in interpreting findings’ was the most frequently cited reason for not performing fundus examinations more regularly in this study. An overall low image-interpretation performance observed in this study is comparable to other Australian-based studies. In two Australian tertiary EDs, sensitivity for detecting urgent pathology using fundus images was 40% [29], while in a rural ED, sensitivity was as low as 29%[30]. However, a US-based study reported diagnostic accuracies of 62.5% for central retinal artery occlusion and 68.4% for papilledema among 54 emergency clinicians[31], which can reflect the need for appropriate ophthalmoscopy training amongst Australian ED specialists and trainees and highlight the need for enhanced training and teleophthalmology support [32].

Male gender, specialist emergency medicine training, willingness to perform fundus examination themselves and higher self-reported confidence in fundus examination are associated with higher odds of accurate image interpretation. The observed association with gender should be interpreted cautiously, as without multivariable adjustment, it is unclear whether gender was independently associated with performance in this cohort. This association may reflect differences in prior exposure or confidence with procedural skills[33, 34], rather than an independent effect of gender itself. Specialist training and willingness to perform examinations likely indicate more hands-on experience. Higher confidence may reflect stronger proficiency arising from training or experience. In contrast, prior fundus examination training in ED and perceived importance of fundus examination were not significantly associated with performance, suggesting that limited training or perception alone may be insufficient to improve skills. As this was a self-administered online survey, responses may also have been influenced by external factors and variable respondent engagement.

Given the fast-paced ED environment, there is a critical need for rapid, user-friendly fundus examination tools. Retinal cameras can offer a wide field of view and generate digital images that facilitate documentation and telemedicine integration [35]. In our survey, a small proportion of emergency clinicians (11/135, 8.1%) reported access to retinal cameras, which is in contrast to a 2024 Canadian survey, which reported none [14]. Many modern devices support non-mydriatic, automated imaging, streamlining workflow. Evidence increasingly supports their value as an addition to fundus examination in emergency departments [36–41]. FOTO-ED study shows that non-mydriatic retinal photography is time-efficient, well-tolerated and improves detection of retinal pathology [6, 42]. Australian studies confirm their feasibility, image quality and diagnostic utility in EDs [29, 30]. In our study, three-quarters of respondents (97/128, 75.8%) agreed that access to portable retinal cameras could enhance fundus examinations in EDs. As technology advances, portable, automated, non-mydriatic devices may support wider adoption and improve examination rates.

Additionally, 46.9% of respondents (60/128) supported AI-assisted image interpretation and clinical recommendations, highlighting opportunities for AI to augment emergency eye care. While some AI models have demonstrated promising performance in detecting ophthalmic emergencies such as optic disc edema and retinal vascular occlusion [43–47], their translation into clinical workflows remains at an early stage. Further research is warranted to evaluate their feasibility, safety and clinical impact to support real-world implementations.

There are several limitations to our study. First, as a self-reported survey, the findings may be subject to response bias, as clinicians with greater interest in ophthalmology or fundus examination may have been more likely to respond. Social desirability bias may also have led respondents to over-report the perceived importance of fundus examination, confidence, or willingness to perform it, while recall bias may have affected responses regarding prior training and usual clinical practice. Second, as the survey was promoted through the ACEM Bulletin, the total number of ED doctors reached was unknown, precluding an accurate calculation of the response rate. Third, although the survey was national in scope, participation was voluntary and the sample was not designed to be statistically representative of all Australian emergency departments. Therefore, the findings should be interpreted as reflecting the views and reported practices of respondents, rather than definitive national practice patterns. Fourth, the relatively small number of regional and remote respondents limits the generalisability of our findings to these settings, where access to ophthalmology services may be more limited and ED-based fundus examination may be particularly important. Recommendations regarding training, equipment access, teleophthalmology or decision-support pathway should be adapted to local service contexts. Future studies should specifically target regional and remote emergency clinicians to better understand local barriers, resource availability and implementation needs. Fifth, although broad remoteness categories were collected, we did not collect site- or city-level identifiers to preserve respondent anonymity. We were therefore unable to assess potential clustering of responses within the same units or regions, which may have influenced estimates of equipment access and practice patterns. As such, the findings should be interpreted as clinician-reported practices rather than institution-level or geographically representative estimates. Future studies could collect de-identified site or regional information to enable pooled analyses and account for clustering while maintaining confidentiality. Sixth, while we included an image-based interpretation task to assess clinicians’ ability to recognise key fundus findings and associated diagnoses, the limited number of test cases may not fully capture the complexity of real-world presentations. In addition, the inclusion of both signs and diagnoses among response options may have introduced some ambiguity. Finally, univariable regression identified factors associated with image interpretation accuracy, but causality cannot be inferred, residual confounding may remain and no formal correction for multiple testing was applied. Therefore, individual *p*-values should be interpreted cautiously and not over-interpreted as confirmatory evidence.

## Conclusion

This study provides insights into emergency clinicians’ experiences and perceptions about fundus examination, highlighting practical and systemic challenges limiting its routine use. Limited equipment access, inadequate training and low confidence contribute to underuse. Targeted education, evidence-based guidelines and fundus cameras may help support more consistent clinically indicated fundus examination and earlier recognition of sight-threatening or life-threatening retinal findings.

## Summary

### What is known about this topic

- Ocular fundus examination is essential for detecting vision- and life-threatening conditions and guiding neuro-ophthalmic triage in emergency settings.
- Previous studies have shown that emergency doctors in the UK, US, and Canada report limited training and low confidence in ophthalmic emergencies.
- No data exist on Australian practices, equipment availability, or clinician capability.

### What this study adds

- This study provides the first national insight into ocular fundus examination in Australian emergency settings, covering training, equipment, clinician practices, perceived barriers/enablers and diagnostic performance.
- This study shows wide variation in how emergency doctors perform fundus examinations, with retinal cameras available in only a few departments, limited training and consistently low diagnostic accuracy and confidence.
- This study identifies actionable improvements—targeted training, portable fundus cameras and clear guidance and workflows—to enhance emergency eye care.

## Supplementary information

Supplementary tables and figures

Supplementary file 1 Questionnaire

## Data Availability

The datasets generated during and/or analysed during the current study are available from the corresponding author on reasonable request.
