# Supplementary material for: Ocular fundus examination in emergency medicine: an Australian survey of equipment, training, current practice, and clinician perceptions
Source: Eye (Lond). 2026 Sep 17;40(16):2641–9. doi: 10.1038/s41433-026-04844-z (PMC13754772; doi:10.1038/s41433-026-04844-z)
Supplement: Supplementary file 1 [file 41433_2026_4844_MOESM1_ESM.docx]

Supplementary Figure 1. Flow diagram of survey participant eligibility and completion across study sections. (ED = emergency department)

Supplementary Figure 2. Frequency of fundus examination consideration across headache presentations (N=128)

Supplementary Table 1. Current practice and perspective of fundus examination among emergency department doctors (N=128)

|  |  | **N (%)** |
| --- | --- | --- |
| **Indications for fundoscopy** | Visual change | 102 (79.7%) |
|  | DBP ≥120mmHg | 24 (18.8%) |
|  | Acute focal neurologic deficit | 38 (29.7%) |
|  | Headache | 76 (59.4%) |
|  | Other | 10 (7.8%) |
|  | None | 17 (13.3%) |
| **Approach to performing fundus examination for patients in the emergency department when indicated.** | I would perform the fundus examination myself. | 65 (50.8%) |
|  | I would refer the patient to another department or location. | 12 (9.4%) |
|  | I would request a colleague from ophthalmology department to conduct the fundus examination | 37 (28.9%) |
|  | I would request it be conducted by a colleague/support staff within the emergency department. | 11 (8.6%) |
|  | Other | 3 (2.3%) |
| **Confidence in fundus examination** | Not confident at all | 39 (30.5%) |
|  | Not very confident | 55 (43.0%) |
|  | Somewhat confident | 31 (24.2%) |
|  | Very confident | 3 (2.3%) |
| **Importance of fundus examination** | No, it is not important | 23 (18.0%) |
|  | Yes, it is moderate important | 61 (47.7%) |
|  | Yes, it is very important | 44 (34.4%) |

DBP= diastolic blood pressure

Data are presented as number and percentage. Multiple responses were allowed where applicable.

Supplementary Table 2. Perceived barriers to and facilitators of fundus examination in the emergency department, by practice region (N=128)

|  |  | **Major city** | **Regional/remote area** | **Total** |
| --- | --- | --- | --- | --- |
|  |  | **N=96** | **N=32** | **N=128** |
| **Reason why fundus examination is not performed more often in the workplace** | I don’t think more fundus examinations are needed beyond current practice | 15 (15.6%) | 2 (6.3%) | 17 (13.3%) |
|  | The findings would not influence my management decisions. | 29 (30.2%) | 7 (21.9%) | 36 (28.1%) |
|  | It is outside the scope of my current position | 5 (5.2%) | 3 (9.4%) | 8 (6.3%) |
|  | Suitable equipment is unavailable | 44 (45.8%) | 16 (50.0%) | 60 (46.9%) |
|  | Inadequate training to use available equipment | 62 (64.6%) | 24 (75.0%) | 86 (67.2%) |
|  | No pupil dilating eye drops are available | 11 (11.5%) | 5 (15.6%) | 16 (12.5%) |
|  | Insufficient time | 51 (53.1%) | 17 (53.1%) | 68 (53.1%) |
|  | Lack of confidence in ability to interpret findings | 81 (84.4%) | 30 (93.8%) | 111 (86.7%) |
|  | Other | 9 (9.4%) | 1 (3.1%) | 10 (7.8%) |
| **Factors that could help perform fundus examinations more frequently in the ED** | Clearer understanding of which patients require fundus examinations | 51 (53.1%) | 14 (43.8%) | 65 (50.8%) |
|  | Training to enhance emergency doctors' fundus examination skills | 72 (75.0%) | 27 (84.4%) | 99 (77.3%) |
|  | Access to a portable retinal camera | 76 (79.2%) | 21 (65.6%) | 97 (75.8%) |
|  | Assistance from an ophthalmology expert | 56 (58.3%) | 17 (53.1%) | 73 (57.0%) |
|  | AI support for image interpretation and/or clinical recommendations | 48 (50.0%) | 12 (37.5%) | 60 (46.9%) |
|  | Other | 9 (9.4%) | 3 (9.4%) | 12 (9.4%) |

Data are presented as number and percentage. Multiple responses were allowed where applicable.

Supplementary Table 3. Free-text responses to the survey question: *“It has been reported that fundus examination is not routinely conducted in emergency departments. What do you believe are the main reasons fundus examination is not performed more often in your workplace?”*

| Theme | Quote |
| --- | --- |
| Insufficient time | - “No time/space to dilate pupils and perform fundoscopy.” |
| No suitable equipment | - “Direct handheld ophthalmoscopes available only, often broken and not where patient is.” - “There are no lenses to use at our slit lamp.” - “The majority of handheld fundoscopies get dropped/cracked and not replaced (this includes wall fixed equipment and there will only be one mobile for the entire department which is often also broken/lost).” - “Training- slit lamps are usually only used for identification of foreign bodies or corneal defects and I've never seen a slit lamp used for fundoscopy.” |
| Findings wouldn’t influence decisions | - “I will persist in visual changes, does not change emergency management in any way in other conditions. Does not change decision to image for emergency may change medical management of non-acute-emergency conditions.” |
| Inadequate training | - “No reliable training provided, skill set doesn't exist with seniors able to supervise juniors learning at current.” - “Trainees/reg/JMOs transient - would need clear scope (much like US training) as when suitably skilled to change management decisions in findings.” |
| Lack of skill and confidence | - “Nobody from ophthalmology would ever trust our findings and is always going to repeat the examinations a couple of hours later. Is that good for the patient? Patient should have it done once and done properly. - just like a speculum exam” - “Not confident to utilise findings to change management.” - “Rarely done and therefore clinicians deskill, difficult to visualise fundus” - “Heavy reliance on imaging to assess for evidence of raised ICP rather than fundoscopy. Performed very infrequency therefore reluctance to rely on findings” - “I don't know how to use the lenses anyway.” |
| ED environment and patient factors | - “Environmental considerations- if a patient makes it into a cube/bedspace it is very hard to dim the light in an individual cube to optimise conditions, more often than not when working out of a bed blocked department no beds/cube spaces are available- patients will often be seen in a corridor or a temporary chair.” - “Patient factors- a lot of patients who I consider may have an acute intracranial pressure rise can be confused or exceptionally photophobic- it can be difficult to position them/tell them thru the fundoscopy process, or very elderly and can't be shifted with lot heavy assistance.” - “I also work in an ED where occupational violence is an issue- on many occasions it is not safe to be that close to someone's face.” |
| Dilation concern | - “Often aiming to DC patients, they often drive self to hospital - dilating pupils will render them unable to drive several hours. ” |
| Outside current job scope/No need more fundus examination | - “Honestly we have enough to do in ED already - I don't want to expand my scope any further. If a patient needs a fundus examination, they need opthal input” - “For fundoscopy looking for chronic changes- ie diabetes/hypertension is generally considered to be outside the scope of ED and routinely performing this would be perceived to be a "waste of time" when departmental pressure is present to move patients through to the most appropriate ward/service etc” - “Optometrists are readily available in the community and have far better equipment. When I'm concerned and discuss with an ophthalmologist, almost all eye emergencies seem suitable for next day consultation. Hence, having the screening performed by an optometrist within 24 hours seems better for the patient.” |

Supplementary Table 4. Free-text responses to the survey question: *“Which of the following factors do you believe could help you perform fundus examinations more frequently in the emergency department?”*

| Themes | Quotes |
| --- | --- |
| Access to a portable retinal camera | - “Retinal cameras would help, but would need to be suitable for use also in patients who cannot sit up.” - “Even having a panoptic ophthalmoscope would be a step up in equipment.” - “Access to appropriate lenses to facilitate fundus examination with a slit lamp” - “Pan optic ophthalmoscopes. Most frequently ophthalmoscopes and auroscopic sets are wall mounted and frequently not working.” - “Having access to appropriate equipment to elicit these findings” |
| Training on fundus examination | - “Reliable training resources. Currently we use you tube!” - “Knowing which findings to seek which would change my immediate management; learning to reliably identify and exclude these” - “We have a portable retinal camera. I refuse to use it - all it will do is document my own malpractice. The photos are not reviewed by anyone, so instead they go into the medical record as clear evidence of me failing to make a diagnosis” |
| Clear understanding of who needs fundus examination | - “Clearer instructions around the indications for/use of dilating drops (ie should we using them to provide a proper opinion in all cases where we think fundoscopy may be useful, or are we actually still expected to look through undilated pupils and give half-arsed opinions?).” - “Many of the reasons we would do fundoscopy are neurological, and require monitoring of pupil size, so we are actively discouraged from using dilating drops in these cases, which means we can't do proper fundoscopy! It's a real Catch-22 situation & one of the reasons almost no emergency clinicians bother with fundoscopy anymore.” |
| More time | - “More time, less patients who also need emergent treatment” - “Time required for dilation and fundoscopy of x patients would currently require researchers to identify which group of patients/conditions they would suggest emergency department staff spend the equivalent amount of less time with. I'm sure the researchers understand the morbidity and mortality attached to current emergency department overcrowding and access block.” |
| Other | - “Don't want to learn to do fundus examinations” - “Researchers spending a day in the emergency department may change their understanding of emergency medicine and management of emergency conditions. The emergency management of headache suspicious for raised intracranial pressure does not involve fundoscopy in 2024. Lack of fundoscopy is not lack of understanding or lack of education/training.” - “I don’t think it needs to be done more frequently - appropriate to refer to outside of ED for vast majority of these presentations, ED isn’t the solution to every medical problem” |

Supplementary Table 5. Logistic regression analysis of factors associated with accurate interpretation of fundus images among ED doctors.

|  | | **N correct/All (%)** | **OR (95%CI)** | **P** |
| --- | --- | --- | --- | --- |
| **Gender** | Female | 132/340 (38.8%) | 1 [Reference] |  |
|  | Male | 124/235 (52.8%) | 1.76 (1.06-2.92) | 0.028 |
| **ED training** | Medical doctor without additional training in emergency medicine | 13/55 (23.6%) | 1 [Reference] |  |
|  | Trainee registrar in emergency medicine | 46/120 (38.3%) | 2.01 (0.82-4.95) | 0.130 |
|  | Emergency medicine fellowship/Overseas trained emergency medicine specialist | 197/400 (49.2%) | 3.14 (1.50-6.55) | 0.002 |
| **Region** | Major city | 181/430 (42.1%) | 1 [Reference] |  |
|  | Regional/remote area | 75/145 (51.7%) | 1.47 (0.80-2.71) | 0.211 |
| **Approach to performing fundus examination** | Referral/Colleague-performed/Others | 99/280 (35.4%) | 1 [Reference] |  |
|  | Self-performed | 157/295 (53.2%) | 2.08 (1.27–3.41) | 0.004 |
| **Confidence** | Not confident at all | 58/175 (33.1%) | 1 [Reference] |  |
|  | Not very confident | 104/245 (42.4%) | 1.49 (0.84–2.63) | 0.171 |
|  | Somewhat confident | 81/140 (57.9%) | 2.77 (1.36–5.63) | 0.005 |
|  | Very confident | 13/15 (86.7%) | 13.11 (1.95–88.37) | 0.008 |
| **Perspective** | No, it is not important | 38/100 (38.0%) | 1 [Reference] |  |
|  | Yes, moderate important | 121/285 (42.5%) | 1.20 (0.62-2.34) | 0.584 |
|  | Yes, it is very important | 97/190 (51.1%) | 1.70 (0.83-3.50) | 0.149 |
| **Received training on fundus examination** | No | 132/320 (41.2%) | 1 [Reference] |  |
|  | Yes | 124/255 (48.6%) | 1.35 (0.82-2.21) | 0.238 |

OR= odds ratios; CI= confidence intervals
