# Supplementary material for: Ocular fundus examination in emergency medicine: an Australian survey of equipment, training, current practice, and clinician perceptions
Source: Eye (Lond). 2026 Sep 17;40(16):2641–9. doi: 10.1038/s41433-026-04844-z (PMC13754772; doi:10.1038/s41433-026-04844-z)
Supplement: Supplementary file 2 [file 41433_2026_4844_MOESM2_ESM.docx]

**Current Practice of Fundus Examination in ED Settings: Survey Study**

Eligibility

1. What is your current country of practice?

- Australia
- New Zealand -> message to say not eligible to participate
- Other -> message to say not eligible to participate

2. What is your highest level of emergency medicine training.

- Emergency Medicine Fellowship/Overseas Trained Emergency Medicine Specialist
- Emergency Medicine Diploma/Advanced Diploma
- Emergency Medicine Certificate
- Trainee registrar in emergency medicine
- Medical doctor without additional training in emergency medicine
- Medical student -> message to say not eligible to participate.
- Not a medical doctor -> message to say not eligible to participate.

3. Are you currently working in a patient-facing clinical role?

- Yes
- No -> message to say not eligible to participate

Demographics

4. What is your current role in the emergency department?

- Consultant emergency physician
- Senior registrar (final years of training in emergency medicine)
- Junior registrar (early training in emergency medicine)
- ED Medical Officer (EDMO)
- Career medical officer (CMO)
- Hospital Medical Officer (HMO)
- Intern

* Role titles may vary across Australian jurisdictions and hospitals. In general, HMO generally refers to a hospital medical officer in postgraduate training; CMO refers to a non-specialist doctor in a career/non-training clinical position; and EDMO refers to a doctor working primarily in an emergency department. Further information on Australian medical career structures is available from NSW health and QLD health: https://www.health.nsw.gov.au/jmo/Pages/glossary.aspx， and https://www.careers.health.qld.gov.au/medical-careers/career-structure.

5. How many years have you practiced in the emergency department?

Please enter the specific number:______year(s).

6. How do you describe your gender?

- Male
- Female
- Non-binary
- Different term
- Prefer not to say

Your institution

*The following questions relate to your primary emergency department of practice. For the purposes of this survey, your primary place of practice is defined as the location where you currently spend the most time.*

7. Would you describe your practice setting as:

- Public
- Private

8. Would you describe the geographical region of your hospital as:

- Major city
- Inner regional
- Outer regional
- Remote
- Very remote

9. How many emergency physicians work in your emergency department in total?

If you are uncertain, please provide an estimate.

10. On average, how many patients visit the emergency department of your hospital on a typical day?

If you are uncertain, please provide an estimate or a range.

11. Please indicate which fundus/retinal examination equipment is readily accessible in the emergency department at your primary place of work (Select all that apply):

- Direct (handheld) ophthalmoscope
- Indirect (head-mounted) ophthalmoscope
- Slit lamp fundoscopy
- Traditional fundus/retinal camera
- Portable retinal camera
- No fundus examination equipment available
- Other, please specify:_______

Your practice

*The following questions relate to any emergency departments where you currently work.*

12. Which types of patients would you consider performing/requesting fundoscopy on? (select all that apply)

- Visual changes
- Diastolic blood pressure of 120mmHg or higher
- Acute focal neurologic deficit
- Headache
- Other, please specify: ___
- None

13. How often would you perform fundoscopy for the following types of patients? (Please use a matrix with options for “Never” “Sometimes” “Always” for each of these.)

- Thunderclap headache
- Progressive headache
- Headache with nausea and vomiting
- Headache with focal neurological symptoms
- Headache aggravated by postures or manoeuvres
- Headache brought on by sneezing, coughing or exercise
- Headache associated with weight loss and/or change in memory or personality
- Headache with fever
- Pregnant women
- Headache onset after 50 years of age
- Past history of increased intracranial pressure

14. If an emergency department patient required fundus/retinal examination, most often:

- I would perform the fundus examination myself.
- I would request it be conducted by a colleague/support staff within the emergency department.
- I would request a colleague from ophthalmology department to conduct the fundus examination
- I would refer to the patient to another department or location.
- Others, please specify: ___

15. How confident are you in performing and interpreting fundus examinations?

- Very confident
- Somewhat confident
- Not very confident
- Not confident at all
- It depends, please specify: ____

16. Have you received any training in fundus examination within the emergency department setting?

- Yes
- No

[If Yes is selected, then display the question 17-19. If No is selected, then skip to question 20]

17. How frequent was your fundus examination training?

- Single session
- Annual training
- Every two to three years
- Every four to five years
- Less frequent than once every five years

18. In which area(s) have you received training in fundus examination? (select all that apply)

- Direct (handheld) ophthalmoscope operation
- Indirect (head-mounted) ophthalmoscope operation
- Slit lamp fundoscopy
- Fundus/retinal camera operation
- Diagnosis based on ophthalmoscope or fundus images
- Other, please specify: ___

19. How was your fundus examination training delivered?

- Practical training (i.e., hands-on learning)
- Observational (e.g., e-learning videos, seminars, text-based learning, etc.)

20. Do you think that it is important for emergency department doctors be able to perform fundus examination?

- Yes, it is very important
- Yes, it is moderately important
- No, it is not important

21. It has been reported that fundus examination is not routinely conducted in emergency departments. What do you believe are the main reasons fundus examination is not performed more often in your work place?

(Select all that apply)

- I don’t think more fundus examinations are needed beyond current practice.
- The findings would not influence my management decisions.
- Other emergency department staff members are responsible for performing fundus examination.
- It is outside the scope of my current position.
- Suitable equipment is unavailable.
- Inadequate training to use available equipment.
- No pupil dilating eye drops are available.
- Insufficient time.
- Lack of confidence in ability to interpret findings.
- Other, please specify____

22. Which of the following factors do you believe could help you perform fundus examinations more frequently in the emergency department? (Please select all that apply)

- Clearer understanding of which patients require fundus examinations
- Training to enhance emergency doctors' fundus examination skills
- Access to a portable retinal camera
- Assistance from an ophthalmology expert
- AI support for image interpretation and/or clinical recommendations
- Other, please specify______

The following six questions relate to your knowledge and ability to interpret fundus findings.

23. To your knowledge, which of the following ocular sign(s) may be accompanied by headache? (Select all that apply)

- Optic disc oedema
- Optic nerve pallor
- Hypertensive retinopathy
- Retinal hemorrhages
- Retinal vascular occlusion
- None of the above

Interpretation of findings

Clarifying note: Questions 24–28 were intended to assess respondents’ recognition and clinical interpretation of key fundus findings. Although the questionnaire wording used “diagnosis”, the response options included both fundus signs and associated diagnoses; therefore, these items were interpreted as an image-interpretation task rather than a definitive diagnostic assessment.

24. Based on the following image, what is the most likely diagnosis?

- Optic disc oedema
- Retinal hemorrhages
- Retinal artery occlusion
- Retinal vein occlusion
- No abnormality detected
- Other
- Unsure

25. Based on the following image, what is the most likely diagnosis?

- Retinal hemorrhages
- Optic disc oedema
- Retinal vein occlusion
- Retinal artery occlusion
- No abnormality detected
- Other
- Unsure

26. Based on the following image, what is the most likely diagnosis?

- Optic nerve pallor
- Optic disc oedema
- Retinal vein occlusion
- Retinal artery occlusion
- No abnormality detected
- Other
- Unsure

27. Based on the following image, what is the most likely diagnosis?

- Retinal hemorrhages
- Optic nerve pallor
- Optic disc oedema
- Retinal vein occlusion
- No abnormality detected
- Other
- Unsure

28. Based on the following image, what is the most likely diagnosis?

- Retinal hemorrhages
- Optic disc oedema
- Retinal vein occlusion
- Retinal artery occlusion
- No abnormality detected
- Other
- Unsure
